# Survival from melanoma of the skin in England and Wales up to 2001

**DOI:** 10.1038/sj.bjc.6604586

**Published:** 2008-09-23

**Authors:** C H Ottensmeier, M Gore

**Affiliations:** 1 Cancer Sciences Division, Southampton University, Tremona Road, Southampton SO16 6YD, UK; 2 Department of Medicine, The Royal Marsden Hospital (NHS Trust), Fulham Road, London SW3 6JJ, UK

## Clinical presentation and diagnosis

Malignant melanoma is a tumour that arises from pigmented cells, the melanocytes. The majority of tumours present as pigmented lesions in the skin and are known as cutaneous malignant melanoma (CMM). Any part of the integument may be involved, and the distribution varies according to gender and race (Cress and Holly, 1997) with the most common presentation being on the trunk for men and the lower limbs for women. According to recent data (Rachet *et al*, 2008) there is an ongoing shift towards trunk melanomas for both genders in the United Kingdom. Melanoma can occur in any age group. Mortality rates peak in people aged 60–74 years but 15% of melanoma deaths occur in people aged less than 50 years (Cancer Research UK, 2008). Childhood melanoma is exceptionally rare.

The main tools used to assess the likelihood of a cutaneous lesion representing a melanoma are the Glasgow seven point checklist and the American ABCD system (McGovern and Litaker, 1992). The transition from benign naevus to melanoma was first described by Clark (Clark *et al*, 1984). The genetic events that have a function in the development of melanoma are increasingly understood (Miller and Mihm, 2006) and this understanding is now also feeding into the development of novel therapeutic strategies. As in other tumours, hereditary genetic characteristics can have an important function in the pathogenesis of melanoma. This is illustrated by the observation that in melanoma families a substantial proportion carry mutations in the CDKN2A tumour suppressor gene (Flores *et al*, 1997; Platz *et al*, 1997; Lang *et al*, 2005).

Critical risk factors for developing CMM include phenotypic factors, in particular skin type (Tsao *et al*, 2004), which affects the risk conferred by environmental exposure to solar radiation (Whiteman *et al*, 2006). The link between tanning and risk for melanoma lies in polymorphisms in the *MC1R* (melanocortin receptor 1) gene, which impair the tanning response to UV light (Miller and Mihm, 2006). Unsurprisingly any individual who has had a melanoma in the past is at increased risk of developing a second primary melanoma. However UV-induced damage is not an absolute requirement for the genesis of melanoma, as melanoma can arise in a wide range of internal organs, including mucosal sites and visceral locations (Thoelke *et al*, 2004). The incidence of noncutaneous melanomas (NCMM) is not as well characterized as for cutaneous primaries, with the exception of ocular melanomas (Damato, 2006). The overall frequency is approximately 5% of all melanomas and work is ongoing to define both the incidence and outcomes of NCMM.

The mainstay of diagnosis in CMM is clinical assessment by an experienced dermatologist, followed by an excision biopsy. Histological assessment may be supplemented by immunohistochemistry for antigens such as S100 and HMB45. S100 belongs to the family of calcium binding proteins and can be detected in almost all benign naevi and malignant melanocytic tumours of the skin, as well as in Langerhans cells in the skin. HMB45 reacts with a neuraminidase-sensitive oligosaccharide side chain of a glycoconjugate, which is present in immature melanosomes and cutaneous melanocytes.

There is a close association between the thickness of the tumour in millimetres and outlook, and this is reflected in the current AJCC staging system (Balch *et al*, 2001). The prognosis for the majority of patients with thin tumours <1 mm is excellent, however, the outlook rapidly worsens as tumours become thicker. Additional adverse features include ulceration of the primary, high mitotic index and invasion of vessels. Ulceration is incorporated into the AJCC staging system. Education of the population in relation to awareness of the risk factors for melanoma, has an important function in prevention and there are data that show this can indeed be beneficial (MacKie *et al*, 2003). A recent advance has been the detection of early lymph node involvement by sentinel node biopsy (SNB). There is no doubt that this staging method picks up stage III patients early (Johnson *et al*, 2006; Kettlewell *et al*, 2006) and leads to stage migration of patients with better prognosis for stage III disease who were regarded as having stage II disease earlier. There is a resultant improvement in survival for both groups. Whether the procedure itself confers a survival advantage and more importantly, whether patients with positive SNB nodes should be offered aggressive surgical management with block dissections remains uncertain. This latter question is the subject of the ongoing large multicenter and multinational MLST II trial.

## Treatment and outlook

The mainstay of treatment for melanoma, CMM or NCMM, remains surgery. Guidelines for width of surgical excision of CMM and follow up are published, reflecting the new AJCC staging system (Roberts *et al*, 2002). Where surgery is not feasible for locally advanced or metastatic disease, palliative radiotherapy can be used. For a few highly selected patients isolated limb perfusion using melphalan without or with tumour necrosis factor can offer local control (Grunhagen *et al*, 2006). No significant progress has been made for the treatment of metastatic disease, where dacarbazine remains a poorly effective standard of care. Multiagent chemotherapy without or with biological response modifiers has shown higher response rates but has not impacted survival (Eggermont, 2006).

A great deal of work has been undertaken in the area of immunotherapy. To date, the results have been disappointing, perhaps because of a lack of understanding of the underlying mechanisms that confer resistance to immunologically based therapies. The most convincing evidence that supports the use of immunotherapeutic strategies comes from data on transfer of autologous tumour infiltrating T cells, although benefit has only been shown in a small number of highly selected patients (Dudley and Rosenberg, 2003). Nevertheless this approach demonstrates the potential effectiveness of immunotherapy and other rational strategies are under investigation, which capitalise on our improved understanding of the tumour immunology in other solid tumours and infectious diseases. Recently anti-CTLA4 antibodies, which are potent activators of immune responses, have been combined with vaccines and this approach may deliver a real improvement in the treatment of advanced disease. However it is also clear that such strategies come at a cost, with significant side effects being common (Beck *et al*, 2006).

In parallel to the use of tyrosine kinase inhibitors in other tumours, in particular renal cell carcinomas, these agents are undergoing investigation in phase III studies in patients with melanoma. Sorafinib mainly targets BRAF but it is also a multitargeting agent. On its own it appears to be of limited clinical use, however combinations with chemotherapy and anti-vascular agents are being explored (Strumberg, 2005). Similarly monoclonal antibody therapies are being explored in melanoma and other solid tumours (reviewed in Adams and Weiner, 2005). Antiangiogenic agents are likely to be a substantial area of investigation in the coming years (reviewed in Jain *et al*, 2006). For instance, Bevacizumab, which confers a survival advantage in colorectal cancer (Meyerhardt and Mayer, 2005) is about to be evaluated in melanoma in the adjuvant setting in the United Kingdom. Even now the role of interferon in the adjuvant setting remains to be defined. While a small survival benefit has become apparent, both dose and duration of treatment with interferon remain uncertain. An interesting new strategy is to target adhesion molecules, such as a-integrins and two such antibodies have recently entered clinical trials. A very different approach is provided by the use of statins either for chemoprevention or in combination with chemotherapy (reviewed in Demierre *et al*, 2005).

Currently, patients with high risk primaries or advanced disease should be offered entry into clinical trials as these are crucial for the identification of promising new strategies in the management of this disease. This research effort will be facilitated by the multidisciplinary team reviews that take place for all patients with malignancy.

## Interpretation of survival patterns against this clinical background

The analysis by Rachet *et al* (2008) in this journal shows that as in other countries (Micheli *et al*, 2002) the rate of incidence of cutaneous melanoma in the United Kingdom is rising more steeply than for most other cancers. This may be related to an increasingly affluent population, which is more frequently exposed to intermittent UV radiation. There also appears to be a narrowing of the deprivation gap in melanoma survival in the United Kingdom and it is likely that the increased awareness of melanoma as a lethal disease is because of this. Increased awareness also results in the detection of thinner tumours with a more favourable outcome (MacKie *et al*, 2003). This explanation is likely, as current treatments have not had a significant impact on either preventing or treating advanced disease. Unless the new treatments currently undergoing clinical investigation show a benefit for thicker, node positive or metastatic tumours, the most important impact on survival from melanoma will be derived from further education and prevention campaigns for the foreseeable future. One example for this is the SunSmart campaign (http://info.cancerresearchuk.org/healthy living/sunsmart/) funded by Cancer Research, United Kingdom and the departments of health, and such initiatives should be supported and expanded.
